# Effects of dietary supplementation with medicinal plant mixtures and immunostimulants on the immune response, antioxidant capacity, and hepatopancreatic health of Chinese mitten crab (*Eriocheir sinensis*)

**DOI:** 10.3389/fimmu.2024.1347736

**Published:** 2024-09-02

**Authors:** Anran Wang, Jie Xu, Xin Zhang, Xuran Liu, Mengge Li, Xiaojing Dong, Shuyan Miao

**Affiliations:** College of Animal Science and Technology, Yangzhou University, Yangzhou, China

**Keywords:** medicinal plants, immunostimulants, immune response, antioxidant capacity, *Eriocheir sinensis*

## Abstract

**Introduction:**

This study aimed to evaluate the efficiency of tea polyphenols (TP) and medicinal plant mixtures (*Astragalus membranaceus* + *Lonicera japonica*, *Rheum officinale* Bail + *Scutellaria baicalensis* + *Platycladus orientalis*) combined with astaxanthin (AST), benzoic acid (BA), and yeast complex on the health status of *Eriocheir sinensis*.

**Method:**

A total of 630 crabs (male crabs: 41.51 ± 1.63 g; female crabs: 47.27 ± 0.79 g) were randomly distributed into seven groups with three replicates (male: female, 1:1). These crabs were fed as follows for 8 weeks: basal diet (M1), M2 (M1 + 100 mg/kg TP), M3 (M1 + 2.0 g/kg *A. membranaceus* + 20 g/kg *L. japonica*), M4 (M1 + 2.5 g/kg *R. officinale* Bail + 1.5 g/kg *S. baicalensis* + 1.0 g/kg *P. orientalis*), and M5, M6, M7 (M2, M3 and M4 with 600 mg/kg AST +1.0 g/kg BA + 20 mg/kg yeast complex added, respectively).

**Results and discussion:**

The results showed that the activities of acid phosphatase (ACP), alkaline phosphatase (AKP), and lysosome (LZM) in the hemolymph were significantly increased in M5, M6, and M7 (*P* < 0.05), and the highest phagocytosis index (PI) and LZM activity were observed in M7 of female crabs. Moreover, the antioxidant indicators superoxide dismutase (SOD), glutathione (GSH), glutathione peroxidase (GPx), and catalase (CAT) of hepatopancreas were also significantly improved in M5, M6, and M7 (*P* < 0.05), while the malondialdehyde (MDA) contents showed an opposite trend. Furthermore, a morphological examination also showed the improved histological structure of hepatopancreas in M7, especially as seen in the clear lumens, no vacuolation, and integrity of the basal membrane of the hepatopancreatic tubule. Taken together, these results suggested that 2.5 g/kg *R. officinale* Bail, 1.5 g/kg *S. baicalensis*, and 1.0 g/kg *P. orientalis* in combination with 600 mg/kg AST, 1.0 g/kg BA, and 20 mg/kg yeast complex could improve the non-specific immunity, antioxidant capacity, and hepatopancreatic health of *E. sinensis.*

## 1 Introduction

The Chinese mitten crab (*Eriocheir sinensis*) is a commercially important freshwater species in China. In 2023, the production of *E. sinensis* reached 888,629 tons in China (1). However, the expansion of the *E. sinensis* industry has resulted in frequent disease outbreaks due to intensive aquaculture practices, leading to impaired immunity and poor oxidative stress response (2). Consequently, the application of specialized antioxidants and immunostimulants is required to boost antioxidant capacity and immune response and prevent pathogenic infections.

*E. sinensis* relies primarily on innate immunity, including cellular and humoral immune responses (3). Therein, hemocytes play a critical role in cellular immunity (4). The total hemocyte count (THC) and phagocytosis index (PI) are important parameters for assessing the cellular defense in crustaceans (5, 6). Moreover, enzymes such as alkaline phosphatase (AKP), acid phosphatase (ACP), and lysosome (LZM) are specific indices for evaluating the humoral immunity responses of *E. sinensis* as they participate in eliminating and hydrolyzing pathogen microorganisms (7–9). Additionally, the antioxidant capacity of the hepatopancreas also reflects the health status of *E. sinensis* (10). The total antioxidant capacity (T-AOC), catalase (CAT), superoxide dismutase (SOD), reduced glutathione (GSH), and glutathione peroxidase (GPx), together with the contents of malondialdehyde (MDA), are commonly used as major indicators for the evaluation of antioxidant capacity and disease surveillance in the hepatopancreas of *E. sinensis* (11–13). These enzymes and indicators play defensive roles against invading diseases and in detoxification in the antioxidant system.

Medicinal plants and their extracts have been used as promising alternatives to antibiotics and immunoprophylactics owing to their high efficacy, low toxicity, and environmentally friendly nature (14). Currently, studies have investigated the application of herbal extracts as feed additives in aquaculture, aimed at promoting growth, modulating immunity, and preventing disease in aquatic animals (15–18). Tea polyphenols (TP), the polyhydroxy phenolic compounds that are extracted from green tea, have been reported to enhance the immune response in fish species (19–21). In addition, Chinese medicinal plants, such as *Astragalus membranaceus*, *Rheum officinale* Bail, or their extracts also have been used as dietary supplements to enhance the immune system and antioxidant capacity of aquatic animals (22–24). Ardo et al. reported that *A. membranaceus* and *L. japonica* extracts (0.1%) enhanced the immunity of Nile tilapia (*Oreochromis niloticus*) (22). In addition, the anthraquinones extracted (0.1%-0.2%) from *Rheum officinale* Bail and *Scutellaria* polysaccharide (150 mg/kg) have also been demonstrated to enhance the immunity and antioxidant capacity of giant freshwater prawn (*Macrobrachium rosenbergii*) (23, 24). Apart from a single supplement of herb or herbal extract, the herbal mixture containing more than two species or combined with commercial products has been shown to boost the immune response of hosts. In the research by Abarike et al., it has been revealed that the combination medicinal plants (*A. membranaceus*, *Angelica sinensis*, *Crataegus hupehensis*) (5 g/kg) with commercial probiotic *Bacillus* (5 g/kg) was more effective than each separately in improving immunity in Nile tilapia (25), which suggested that the combination of herbs and probiotic provided a synergistic effect on the immune response.

Astaxanthin (AST), an oxidized form of β-carotene, is abundant in the carapace of crustaceans and marine environments (26). Studies have reported that dietary AST has benefits for *E. sinensis*, including non-specific immunity and antioxidant capability (11, 27, 28). Benzoic acid (BA) (0.1%) also has been used as a growth promotor in Nile tilapia (29). Moreover, it has been reported that yeast can act as an immunostimulant and antioxidant in shrimp aquaculture because of its nutritional value (30). Zhang et al. demonstrated that dietary yeast extract (5 g/kg) improved the immunity and antioxidant status of *E. sinensis* (31). However, the combined effect of AST, BA, and yeast complex on the antioxidant and immune response of *E. sinensis* has not been reported. Therefore, developing a feed additive with an optimal combination and dosage of medicinal plants and immunostimulants is essential to maintain the health of *E. sinensis.* In the present study, we designed six different combinations of medicinal plant mixtures with or without AST, BA, and yeast complex to evaluate the optimal formulation of feed additive for *E. sinensis* health, which is characterized by the innate immunity, antioxidant capacity, and hepatopancreatic histomorphology of *E. sinensis*. This study will provide a theoretical foundation for using medicinal plants as feed additives for *E. sinensis* aquaculture.

## 2 Materials and methods

### 2.1 Experimental diets

The formulation of the basal diet (M1) is shown in **Table 1**. Soybean meal, peanut meal, and rapeseed meal were chosen as the primary protein sources. Fish oil, shrimp paste, and soybean phospholipid oil were chosen as the primary lipid sources. The proximate composition of the basal diet was analyzed according to the method of Xu et al. (32). Three experimental diets, M2, M3, and M4, were produced by adding 100 mg/kg TP, 2.0 g/kg *A. membranaceus* + 20 g/kg *L. japonica*, and 2.5 g/kg *R. officinale* Bail + 1.5 g/kg *S. baicalensis* + 1.0 g/kg *P. orientalis*, respectively. Then, 600 mg/kg AST + 1.0 g/kg BA + 20 mg/kg yeast complex were added to M2, M3, and M4 diets to produce another three experimental diets named M5, M6, and M7, respectively (**Table 2**). All medicinal plants were supplied by Zhixin Pharmaceutical Co., Ltd., Nanjing, China. AST (purity > 98%) and BA (purity > 98%) were purchased from Yuanye Biotechnology, Shanghai, China. Yeast complex (also named as yeast extracts) was purchased from Angel Yeast Co., Ltd., Yichang, China.

**Table 1:** Ingredient formulation and proximate composition of the basal diet.

Ingredient (g/kg diet)	Content
Fish meal (60% crude protein)	40.00
Chicken meal (65% crude protein)	40.00
Enzymatic feather meal (82% crude protein)	50.00
Soybean meal (46% crude protein)	220.00
Peanut meal (46% crude protein)	200.00
Rapeseed meal (36% crude protein)	150.00
DDGS (26% crude protein)	40.00
Glutamic acid residue (95% crude protein)	30.00
Shrimp paste	20.00
Wheat flour	130.00
Fish oil	10.00
Soybean phospholipid oil	15.00
Bentonite	25.70
Calcium dihydrogen phosphate	10.00
Complex antioxidant	0.30
Calcium propionate	1.00
Lysine (70%)	5.00
Premix^1^	10.00
Guar gum	3.00
*Proximate analysis (% dry weight)*	
Crude protein	40.25
Crude lipids	6.03
Moisture	10.82

DDGS, distillers dried grains with soluble.

^1^Mineral premix (per 1 kg diet): Ca(H_2_PO_4_)_2_, 10 g; MgSO_4_·7H_2_O, 2.4 g; KCl, 4.5 g; NaCl, 2.1 g; FeSO_4_·H_2_O, 155 mg; CuSO_4_·5H_2_O, 40 mg; ZnSO_4_·H_2_O, 80 mg; Mn·SO_4_·H_2_O, 30 mg; KI, 11.7 mg; CoCl_2_·6H_2_O, 4.8 mg; Na_2_SeO_3_, 2.4 mg.

Vitamin premix (per 1 kg diet): Vitamin A, 10000 IU; Vitamin D, 2500 IU; Vitamin K, 64 mg; Thiamine, 60 mg; Riboflavin, 250 mg; Pyridoxine, 60 mg; Calcium pantothenate, 240 mg; Folic acid, 12 mg; Biotin, 50 mg; Cyanocobalamin, 4 mg.

**Table 2:** The design of seven experimental diets for *E. sinensis*.

Groups	Diets
M1	Basal diet
M2	Basal diet +100 mg/kg TP
M3	Basal diet +2.0 g/kg *A. membranaceus* + 20 g/kg *L. japonica*
M4	Basal diet +2.5 g/kg *R. officinale* Bail + 1.5 g/kg *S. baicalensis* + 1.0 g/kg *P. orientalis*
M5	Basal diet +100 mg/kg TP + 600 mg/kg AST + 1.0 g/kg BA + 20 mg/kg yeast complex
M6	Basal diet +2.0 g/kg *A. membranaceus* + 20 g/kg *L. japonica* + 100 mg/kg TP + 600 mg/kg AST + 1.0 g/kg BA + 20 mg/kg yeast complex
M7	Basal diet +2.5 g/kg *R. officinale* Bail + 1.5 g/kg *S. baicalensis* + 1.0 g/kg *P. orientalis* + 600 mg/kg AST + 1.0 g/kg BA + 20 mg/kg yeast complex

TP, tea polyphenols; AST, astaxanthin; BA, benzoic acid.

Diets were prepared using the method described by Li et al. (33). All dry ingredients were finely ground using a pulverizer (HK820, Xulang Machinery Manufacturing Co., Ltd., Guangzhou, China) and then passed through a 100-mesh sieve. After thoroughly mixing the dry ingredients, the fish oil, soybean phospholipid oil, and distilled water were added to form a dough, and subsequently extruded into 1.5 mm diameter pellets by a feed mill (F-26(II), South China University of Technology Science and Technology Industrial Plant, Guangzhou, China). All diets were dried at 50°C and stored at -20°C.

### 2.2 Experimental animal and conditions

Healthy *E. sinensis* was provided by Jiangsu Haorun Group, Taizhou, China. Prior to beginning the feeding trial, crabs were fed the basal diet three times a day for 2 weeks. Then, 315 male crabs (initial weight: 41.51 ± 1.63 g) and 315 female crabs (initial weight of: 47.27 ± 0.79 g) in the same molting cycle were stored individually in each bucket (0.48 × 0.38 × 0.36 m, L: W: H) to prevent them from fighting each other, and then randomly divided into 7 groups. Each group has three replicates with 30 individuals per replicate (male: female, 1:1). The 30 buckets of each replicate were arranged along the sides of outdoor cement ponds (5.0  ×  5.0  m, L: W).

The crabs were fed with experimental diets at a ratio of 2% of their average body weight twice daily (7:30, 17:00) for 8 weeks. The water temperature was 26 ± 2°C, the dissolved oxygen concentration was above 6.7-7.2 mg/L, and nitrite-N and total ammonia nitrogen were below 0.09 mg/L and 0.3 mg/L, respectively.

### 2.3 Samples collection and analysis

After the 8-week feeding trial, the crabs were sampled for hemolymph and hepatopancreas after 24 h fasting. The hemolymph sampling used the method Miao et al. described (34). Briefly, one part of the hemolymph samples was collected using a 1.0 mL syringe without anticoagulants from the base of the third pereiopod, stored at 4°C overnight, and centrifuged at 8000 r/min for 10 min. Then the serum was collected to analyze the activities of immune-related enzymes, including AKP, ACP, and LZM. Another part of the hemolymph samples was collected using a syringe with an anticoagulant solution (volume 1:1) for the total hemocyte count (THC) and phagocytic index (PI) determination.

One part of the hepatopancreas samples was obtained for antioxidant indices determination and preserved in Trizol regent for RNA extraction. Another part of hepatopancreas samples was stored in Bouin’s fixative for histology analysis.

The THC was determined according to the method described by Zhao et al. (35). Briefly, the uncoagulated hemolymph was fixed with fixative solution (volume 1:1, ingredients: 0.10 M sodium cacodylate and 1.5% glutaraldehyde) for 30 min to measure THC using a hemocytometer under an optical microscope (Olympus DP26, Japan). The total number of hemocytes in the square of the four corners was counted.

Cell density = (total of 4 large cells/4) ×10^5^ cells/L

The phagocytosis index (PI) was determined according to the method used by Zhang et al. (36). The uncoagulated hemolymph was diluted saline (volume 1:1). The phagocytic activity was measured by mixing the *Staphylococcus aureus* suspension with hemolymph at a ratio of 10:1. The final concentration of *S. aureus* was 10^8^ CFU/mL.

PI (%) = (the total number of golden grapes in phagocytes/number of active hemocytes involved in phagocytosis) × 100%

The immune-related enzymes activities of ACP, AKP, and LZM in hemolymph, and the antioxidant indices of hepatopancreas, including T-AOC, SOD, GPx, GSH, CAT, and MDA were measured by commercial kits according to the manufacturer’s instructions (Nanjing Jiancheng Bioengineering Institute, Nanjing, China).

After 24 h of fixation, the hepatopancreas samples were embedded in paraffin, sectioned. After staining with hematoxylin and eosin (H&E), the samples were observed and photographed using an Olympus BX-50 fluorescence microscope (Olympus DP26, Japan).

The relative mRNA level of genes (*proPO* and *crustin*) was analyzed by real-time *q*PCR analysis. Total RNA of hepatopancreas samples was extracted by the Trizol method. 1 μg RNA was taken from each sample and transformed into cDNA using TransScript All-in-One First-Strand cDNA Synthesis SuperMix for *q*PCR (Transgen Biotech, Beijing, China). ChamQ SYBR *q*PCR Master Mix (Vazyme, Nanjing, China) was used for RT-*q*PCR. The detailed methods were described by Li et al. (33). The CDS sequences of the target genes were acquired from the National Center for Biotechnology Information (NCBI), and the primers were designed using Primer Premier 6. The primers were listed in **Table 3**, and *β-actin* was used as the reference gene. All data was analyzed according to the 2^-ΔΔCT^ method.

**Table 3:** Primers used for the real-time qPCR analysis of the genes in *E. sinensis*.

Primer name	Primer sequence (5’ to 3’)
*β-actin*	F: ACCTCGGTTCTATTTTGTCGG R: ATGCTTTCGCAGTAGTTCGTC
*proPO*	F: GACTGTGAAAGCGGCCCTTAGTA R: TTGCTTCCCATTTGCTTCTGTTG
*crustin*	F: GCTCTATGGCGGAGGATGTCA R: CGGGCTTCAGACCCACTTTAC

### 2.4 Statistical analysis

The statistical analyses were performed using SPSS 18.0 (SPSS Inc., Chicago, IL, USA). All results were presented as the means ± standard deviation (S.D.) of three replicates. Comparisons among groups were analyzed by one-way analysis of variance (ANOVA) with Duncan’s test. The statistical significance was considered when *p* < 0.05.

## 3 Results

### 3.1 Hematologic non-specific immune parameters of crabs

In male crabs, the THC was significantly increased in medicinal plant treatment groups (M2, M3, M4, M5, M6, and M7) compared with M1, and reached the highest in M4 and M7 (*p* < 0.05), and the PI was significantly improved in M5 and M7 compared with other groups (*p* < 0.05). In female crabs, the THC also significantly increased in M3, M4, M5, M6, and M7 compared with M1 (*p* < 0.05). Moreover, the PI in M5 and M7 was significantly increased compared to M2 and M7, respectively (*p* < 0.05) (**Table 4**).

**Table 4:** Hematological parameters of *E. sinensis* fed with different experimental diets.

	Male crabs		Female crabs	
	THC (10^5^/L)	PI (%)	THC (10^5^/L)	PI (%)
M1	48.33 ± 0.76^a^	4.47 ± 0.06^a^	48.67 ± 1.26^a^	4.65 ± 0.09^a^
M2	51.50 ± 2.29^b^	5.03 ± 0.08^b^	51.50 ± 2.00^ab^	4.75 ± 0.04^ab^
M3	53.83 ± 2.57^bc^	4.91 ± 0.07^b^	54.50 ± 1.32^b^	4.85 ± 0.08^bc^
M4	55.50 ± 2.18^c^	4.55 ± 0.20^a^	54.33 ± 0.76^b^	4.99 ± 0.10^cd^
M5	54.00 ± 0.87^bc^	5.49 ± 0.07^c^	53.67 ± 2.57^b^	5.15 ± 0.07^de^
M6	54.33 ± 1.04^bc^	5.01 ± 0.08^b^	52.17 ± 1.76^b^	4.97 ± 0.13^c^
M7	55.33 ± 0.76^c^	5.37 ± 0.12^c^	54.00 ± 1.50^b^	5.21 ± 0.16^e^

THC, total hemocyte count; PI, phagocytosis index.

Data in the same column with different superscript letters are significantly different (*p* < 0.05) as determined by Duncan’s test. The values are the means ± S.D. (n = 3).

**Figure 1:**
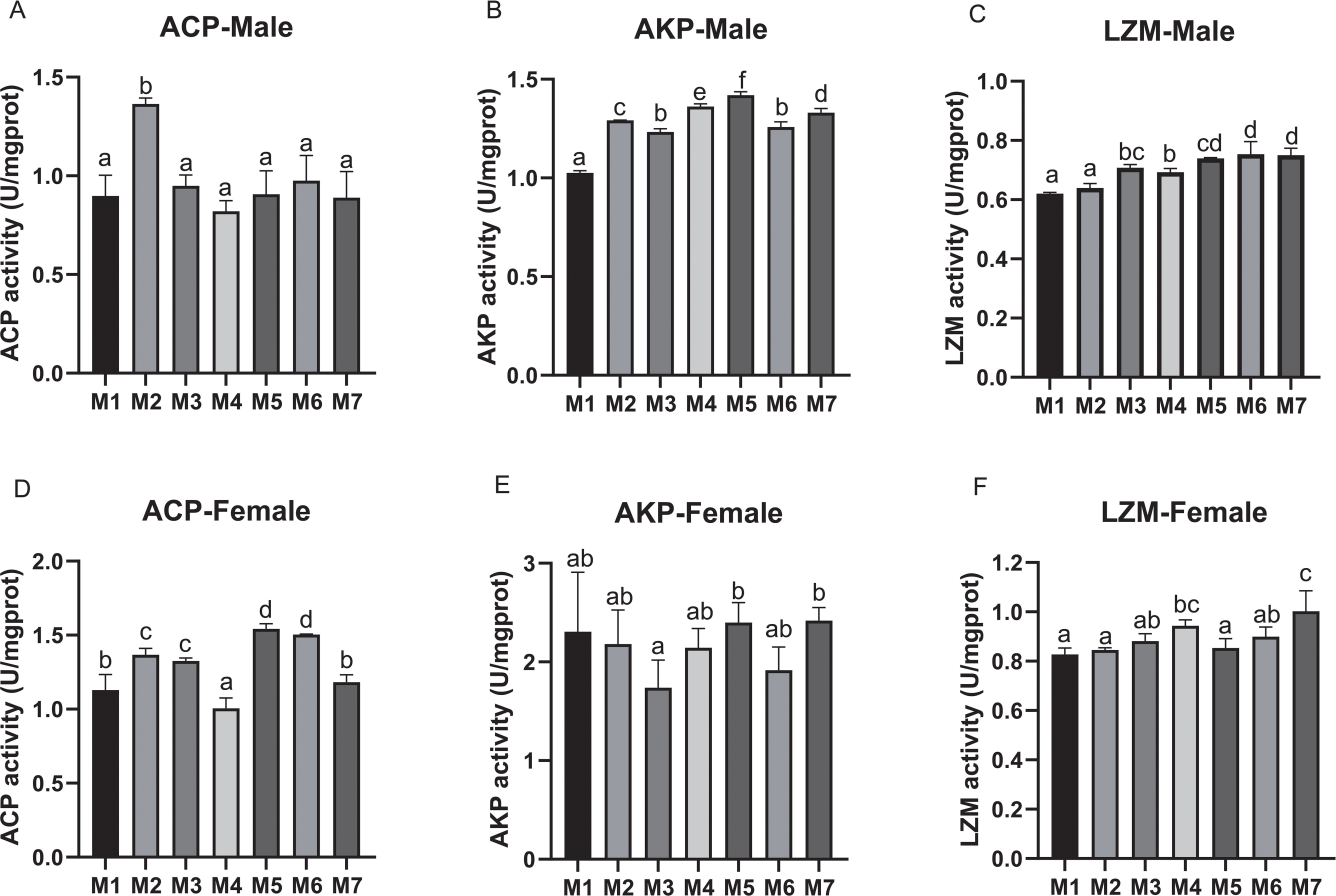
Hematologic immune-related enzyme activity of *E. sinensis* fed seven experimental diets (means ± S.D., n = 3). **(A)** Acid phosphatase (ACP) activity of male crabs; **(B)** Alkaline phosphatase (AKP) activity of male crabs; **(C)** Lysosome (LZM) activity of male crabs; **(D)** ACP activity of female crabs; **(E)** AKP activity of female crabs; **(F)** LZM activity of female crabs. Bars with different letters indicate significance among the treatments (*p* < 0.05).

Furthermore, we examined the activities of ACP, AKP, and LZM in hemolymph. In male crabs, the activity of ACP was significantly increased in M2 compared with other treatments (*p* < 0.05). The activity of AKP was significantly increased in medicinal plant treatment groups (M2, M3, M4, M5, M6, and M7) compared with M1 (*p* < 0.05), and the highest AKP activity of M5 was significantly increased compared to M2. For the LZM activity, it was significantly increased in M3, M4, M5, M6, and M7 compared with M1, as well as significantly increased in the AST, BA, and yeast complex treatment groups (M5, M6, and M7) compared with M2, M3, and M4, respectively (*p* < 0.05) (**Figures 1A–C**).

In female crabs, the activity of ACP was significantly increased in M2, M3, M5, and M6 compared with others (*p* < 0.05), and the ACP activity in M5 and M6 was significantly increased compared to M2 and M3, respectively. Moreover, the activity of LZM was significantly increased in M4 and M7 when compared with others (*p* < 0.05), reaching the highest in M7 (**Figures 1D–F**).

### 3.2 Immune-related genes of hepatopancreas

**Figure 2:**
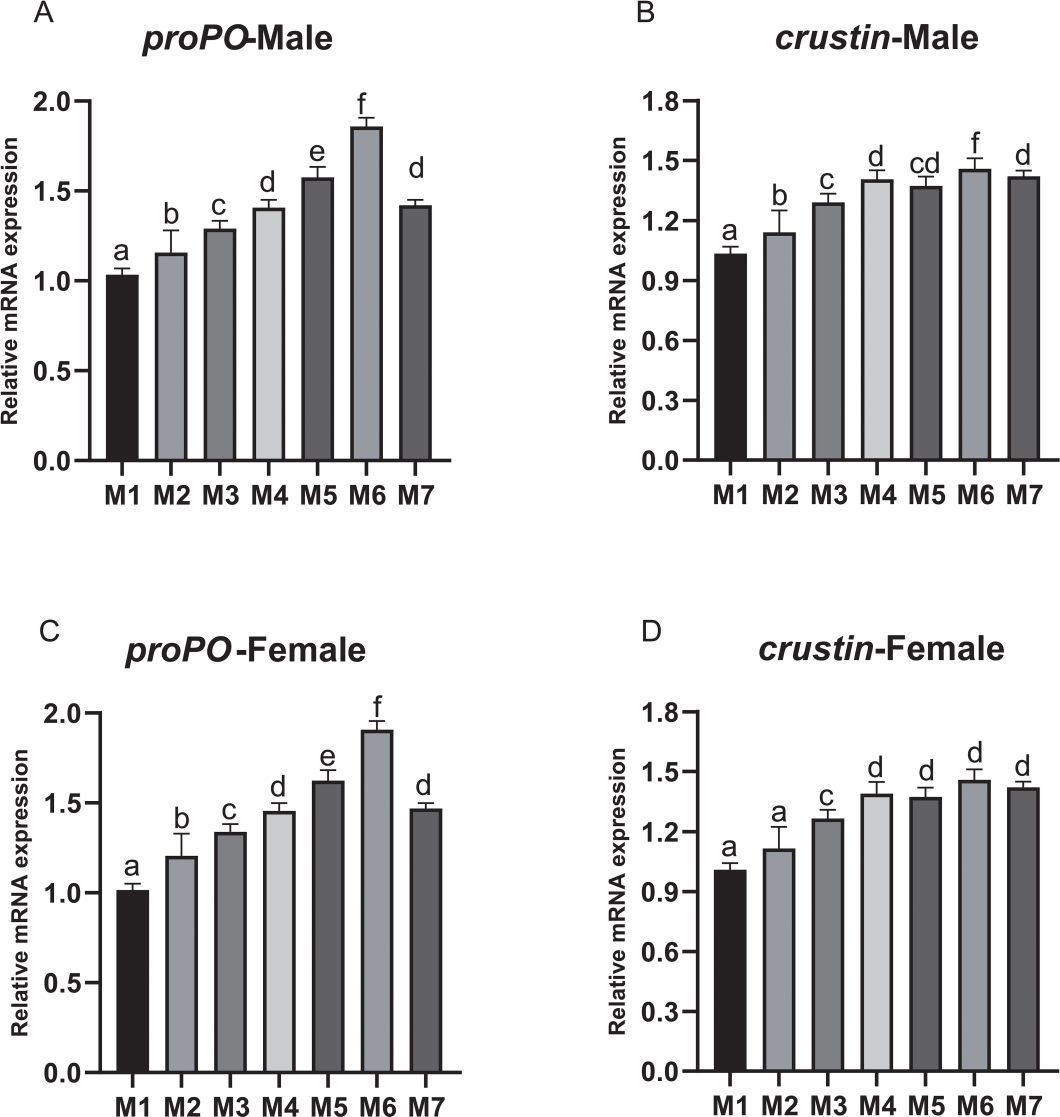
The relative expression levels of *proPO* and *crustin* in hemolymph of *E. sinensis* fed seven experimental diets (means ± S.D., n = 3). **(A)** Prophenoloxidase (*proPO*) of male crabs; **(B)** *crustin* of male crabs; **(C)** *proPO* of female crabs; **(D)** *crustin* of female crabs. Bars with different letters indicate significantly among the treatments (*p* < 0.05).

The *proPO* expression showed an increasing trend from M2 to M6 and reached the highest in M6 (**Figures 2A, C**). Similarly, the expression of *crustin* also showed an increasing trend from M2 to M7, which was significantly higher in M2-M7 for male crabs and M3-M7 for female crabs (*p* < 0.05) (**Figures 2B, D**).

### 3.3 Antioxidant capacity of hepatopancreas in crabs

**Figure 3:**
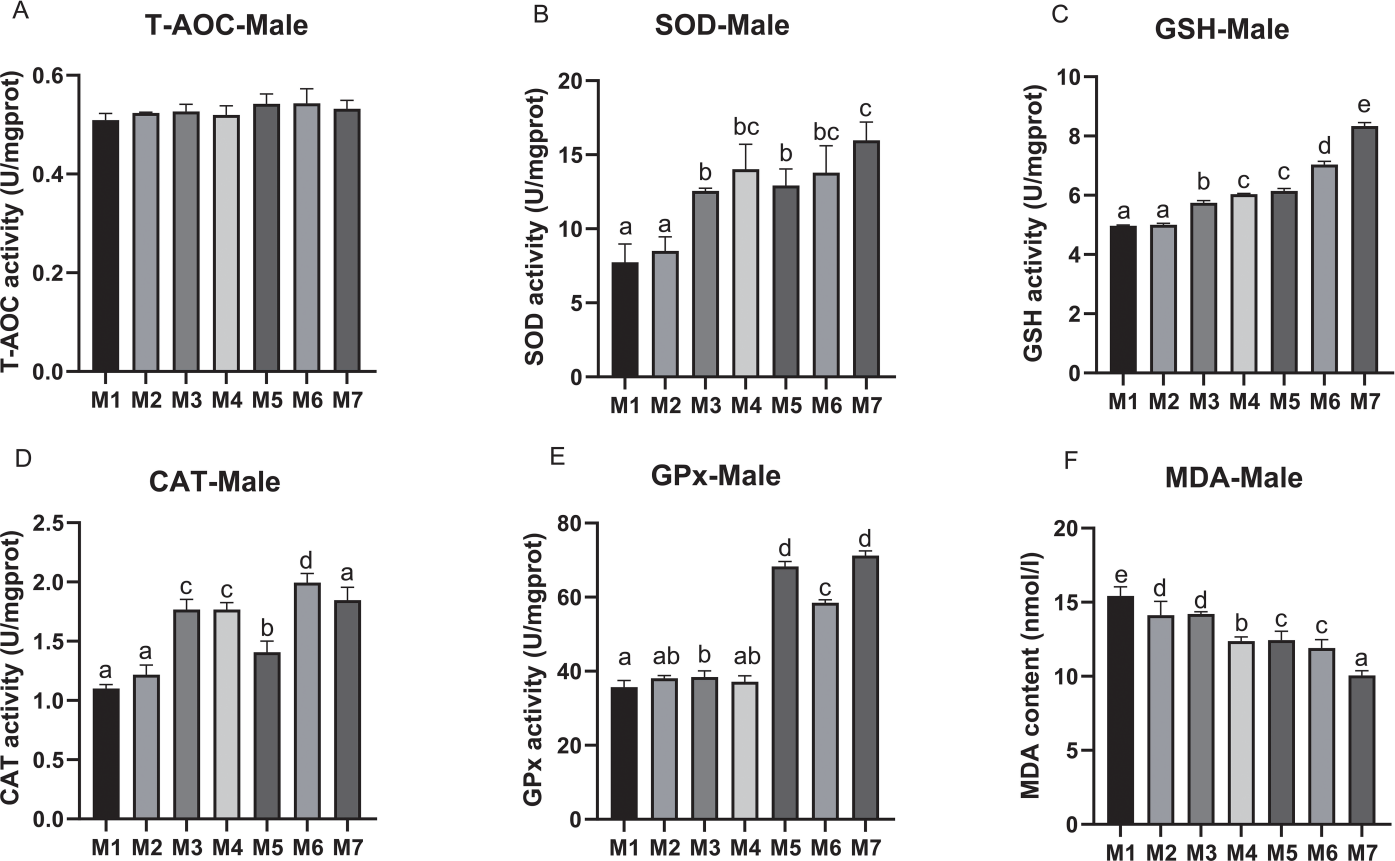
The hepatopancreatic antioxidant parameters of male *E. sinensis* fed seven experimental diets (means ± S.D., n = 3). **(A)** Total antioxidant capacity (T-AOC); **(B)** Superoxide dismutase (SOD); **(C)** Glutathione (GSH); **(D)** Catalase (CAT); **(E)** Glutathione peroxidase (GPx); **(F)** Malondinaldehyde (MDA). Bars with different letters indicate significance among the treatments (*p* < 0.05).

To evaluate the antioxidant capacity of crab hepatopancreas, we measured the antioxidant indices, including T-AOC, SOD, GSH, CAT, GPx, and MDA. In male crabs, no significant difference in T-AOC was found among all treatment groups. The activity of SOD was significantly improved in crabs receiving M3, M4, M5, M6, and M7 compared with M1 and M2, and the SOD activity in M7 was significantly higher compared with other groups (*p* < 0.05). A similar trend was also found in the GSH contents and CAT activities, which were significantly increased in M5, M6 and M7 when compared to M2, M3, and M4, respectively (*p* < 0.05), and reached their highest level in M7 and M6, respectively. The activity of GPx was significantly higher in M5, M6, and M7 than that of other groups, and reached the highest in M7 (*p* < 0.05). Correspondingly, the MDA content was significantly reduced in crabs receiving medicinal plant treatments (M2, M3, M4, M5, M6, and M7) compared to the control. Furthermore, the MDA value in the groups receiving the combined AST, BA, and yeast complex (M5, M6, and M7) was significantly decreased compared with M1-M4, and the lowest was in M7 (*p* < 0.05) (**Figure 3**).

**Figure 4:**
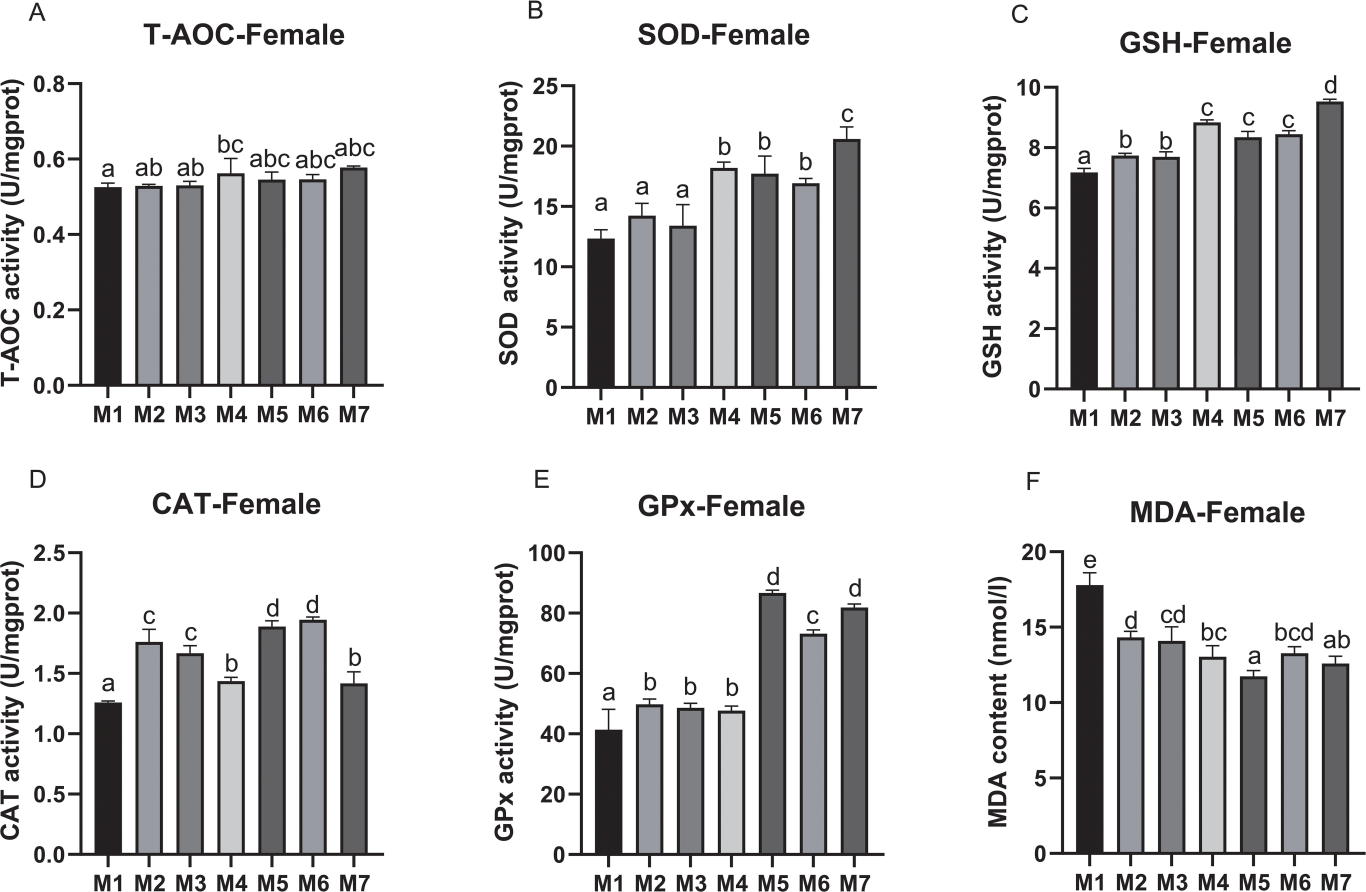
The hepatopancreatic antioxidant parameters of female *E. sinensis* fed seven experimental diets (means ± S.D., n = 3). **(A)** Total antioxidant capacity (T-AOC); **(B)** Superoxide dismutase (SOD); **(C)** Glutathione (GSH); **(D)** Catalase (CAT); **(E)** Glutathione peroxidase (GPx); **(F)** Malondinaldehyde (MDA). Bars with different letters indicate significance among the treatments (*p* < 0.05).

In female crabs, the T-AOC activity was significantly increased in M4 compared to M1(*p* < 0.05), while other groups did not show a significant difference. The activity of SOD in M5, M6, and M7 was significantly increased compared to M2, M3, and M4, respectively, and reached the highest in M7 (*p* < 0.05). For the GSH contents, it was significantly increased in medicinal plant treatment groups (M2, M3, M4, M5, M6, and M7) compared to the control. Moreover, the M5, M6, and M7 treatment groups had significantly higher GSH contents than M2, M3, and M4, respectively, and M7 showed the highest GSH contents (*p* < 0.05). For the activity of GPx, it was significantly increased in M5, M6, and M7 compared with other groups, and the highest was found in M5 (*p* < 0.05). As compared to M2 and M3, M5 and M6 had higher CAT activity (*p* < 0.05). Similarly, the MDA level in crabs in the medicinal plant treatment groups (M2, M3, M4, M5, M6, and M7) was also significantly decreased compared to M1, and M5 showed a significantly lower MDA value than M2 (*p* < 0.05) (**Figure 4**).

### 3.4 Hepatopancreas histology

**Figure 5:**
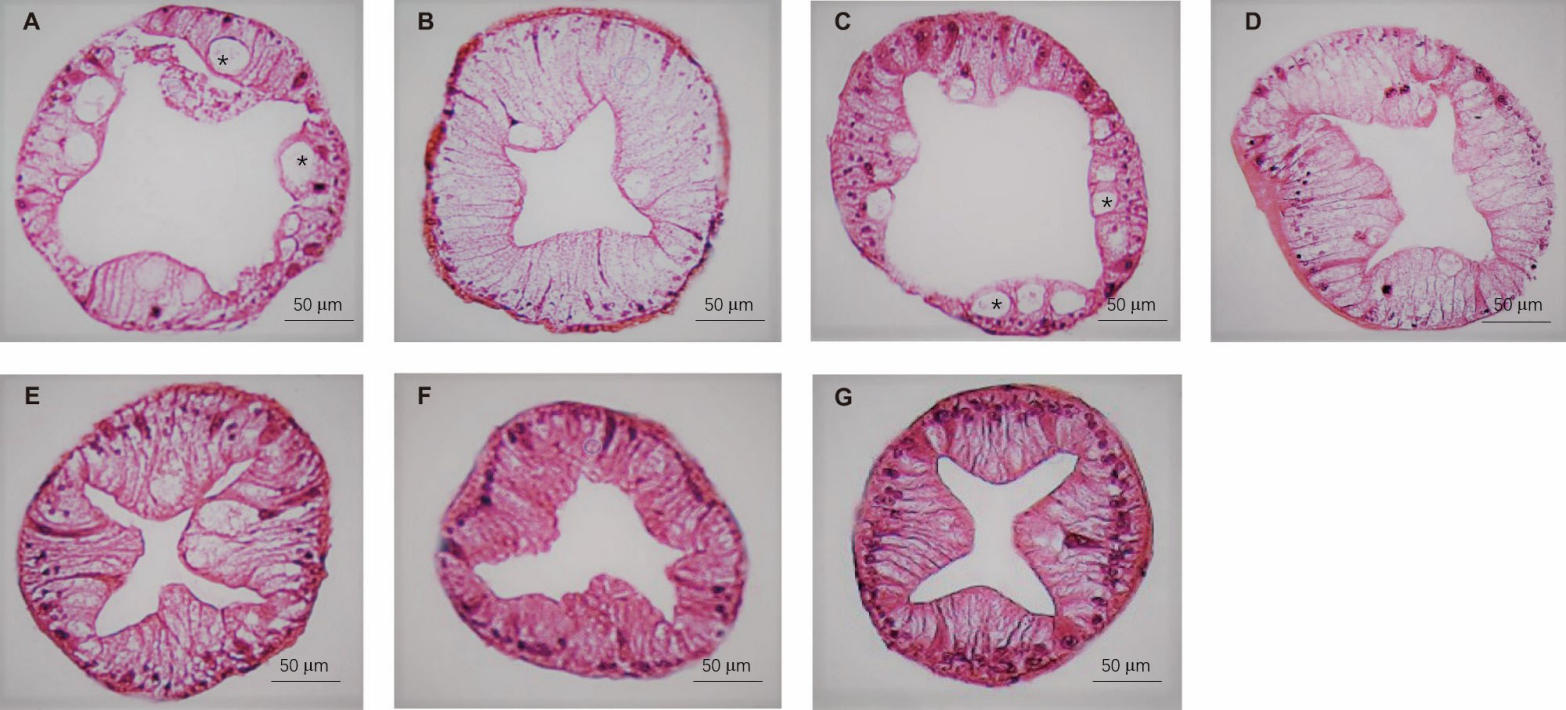
The hepatopancreas histological structure of male crabs fed seven experimental diets. **(A:** M1, **B:** M2, **C:** M3, **D:** M4, **E: **M5, **F:** M6, and **G:** M7). The asterisk indicates vacuoles within the hepatic tubules.

**Figure 6:**
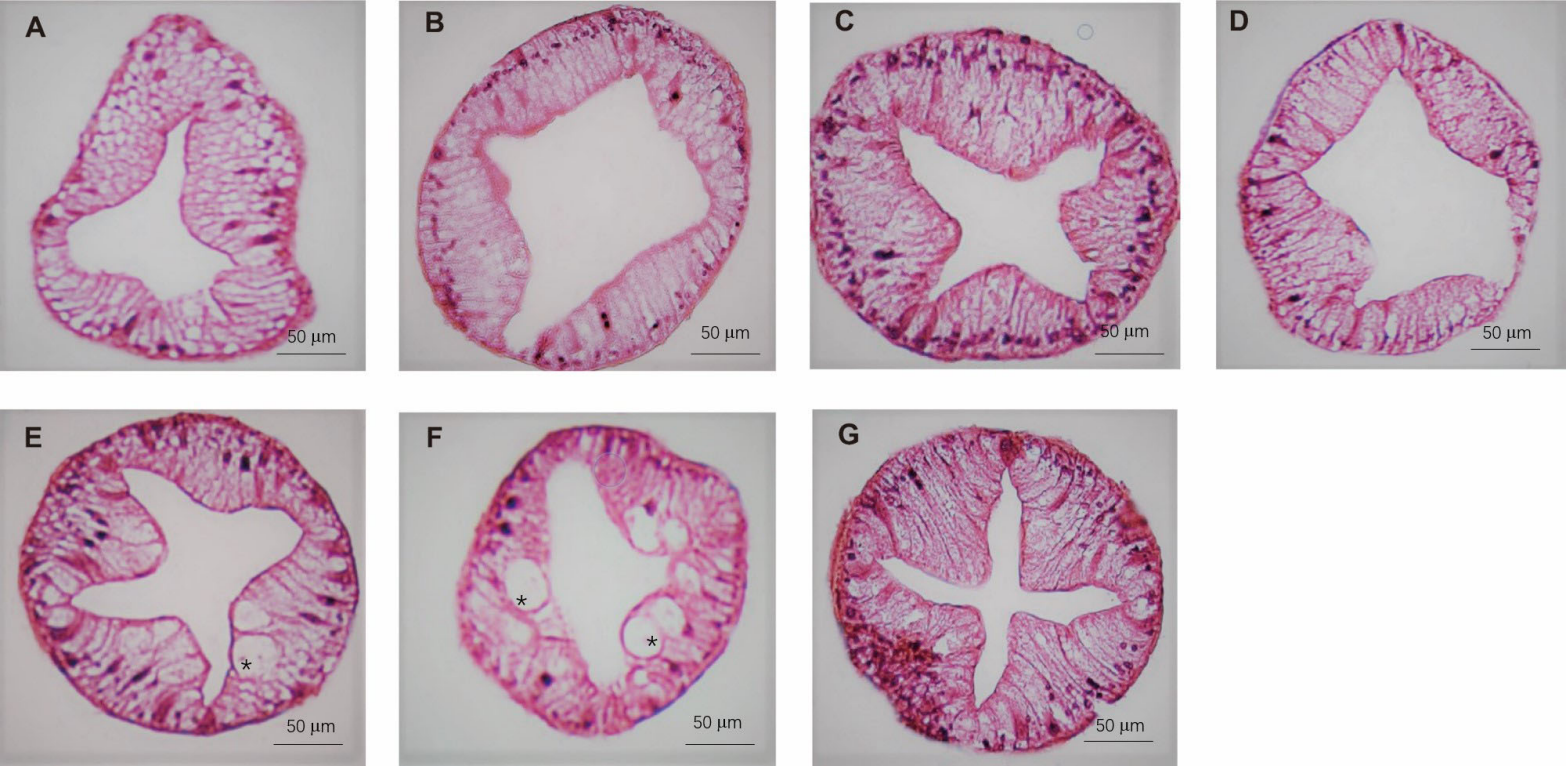
The hepatopancreas histological structure of female crabs fed seven experimental diets. **(A: **M1, **B: **M2, **C:** M3, **D: **M4, **E:** M5, **F:** M6, and **G:** M7). The asterisk indicates vacuoles within the hepatic tubules.

The histology of the crab hepatopancreas was examined by H&E staining. In male crabs, the epithelial cells of hepatopancreas were highly vacuolated in M1 and M3, and slight vacuolation appeared in the M2, M4, and M5 groups. The vacuolar structures disappeared in M6 and M7, and the basal laminae of hepatopancreas were thickened in M5, M6, and M7 compared with M1, M2, M3, and M4 (**Figure 5**). In female crabs, the basal laminae of hepatopancreas were separated with honeycomb-like pores in M1, while the hepatopancreas exhibited a few vacuoles in M5 and M6. Collectively, the hepatopancreas of both male and female crabs in M7 showed clear lumens with no vacuolation and an intact basement membrane (**Figure 6**).

## 4 Discussion

Medicinal plants, including *A. membranaceus*, *L. japonica*, *R. officinale* Bail, *S. baicalensis*, and *P. orientalis*, have been applied as feed additives for aquatic animals. They can be used individually in a mixture, or in combination with other immunostimulants. In many aquatic species, studies have demonstrated that the combination of medicinal plants improves the immune response better than a single additive (25, 37, 38). In the current study, we evaluated the efficacy of dietary single (TP) or medicinal plant mixtures (*A. membranaceus* + *L. japonica*, *R. officinale* Bail + *S. baicalensis* + *P. orientalis*) in combination with AST, BA, and yeast complex or not, for the innate immunity, antioxidant capacity, and histomorphology of hepatopancreas *E. sinensis*.

Due to the lack of a specific immune system, hemocytes play a crucial role in crustaceans’ immune response (39). Phagocytosis is an important cellular defense reaction against invading pathogens (40). Ardó et al. found that feeding tilapia with *A. membranaceus* and *L. japonica* significantly enhanced phagocytic activity (22). Moreover, studies also showed that dietary supplementation with *R. officinale* extract and *S. baicalensis* have a positive effect on the phagocytic activity of orange-spotted grouper (*Epinephelus coioides*) and red drum (*Sciaenops ocellatus*), respectively (41, 42). In alignment with these results, the present study has shown that PI was significantly higher in the combined medicinal plant treatment groups (M2 and M3 for male crabs, M3 and M4 for female crabs) than in the M1 group. Furthermore, we observed that the PI of M5 and M7 was significantly increased compared with M2 and M4, respectively. AST, BA, and yeast complex have been applied as immunostimulants to improve the immune status of aquatic animals (11, 27–29, 31). Studies have reported that dietary AST (50 mg/kg) increased the phagocytic activity of Pacific white shrimp (*Penaeus vannamei*) (43) and Asian seabass (*Lates calcarifer*) (44). Moreover, Abu-Elala et al. demonstrated that dietary 0.2% yeast cell wall supplementation improved the PI of Nile tilapia (45). These results suggested that a combination of medicinal plant mixtures with AST, BA, and yeast complex would augment the phagocytic activity of crabs.

ACP and AKP are two important nonspecific phosphohydrolases that can destroy and eliminate invading bacteria and are of great importance in maintaining the health of crustaceans. LZM is a bactericidal factor that plays a key role in preventing pathogenic bacteria by degrading the peptidoglycan layer in their cell walls (46). In this study, we observed that the activities of hemolymph ACP in female crabs and the LZM activity in male crabs were significantly increased in M5, M6, and M7 treatment groups compared with M2, M3, and M4, respectively, and the highest LZM activity was found in M7 for female crabs. Consistently, the effects of chemical ingredients from *Scutellaria* and *Rheum officinale* Bail on enhancing ACP, AKP, and LZM activities have been reported in the study of giant freshwater prawn (*M. rosenbergii*) (23, 24). In *E. sinensis*, studies have also demonstrated that dietary AST or yeast improves the ACP, AKP, and LZM activities (27, 47). These results demonstrated that medicinal plant mixture (*R. officinale* Bail, *S. baicalensis*, *P. orientalis*) combined with AST, BA, and yeast complex boost the immune response of crabs. Thus, it can be inferred that the improved immune status in crabs may be attributed to the consequence of synergy among the active molecules contained in the medicinal plants (e.g. anthraquinones, polysaccharides) and immunostimulants. However, the mechanisms or modes of action of these effective ingredients in crabs require further investigation. The proPO system and Crustin are major components in the innate immunity of *E. sinensis* (48, 49), especially participating in defense against pathogens. The results showed that the expression levels of *proPO* and *crustin* were significantly increased in the M5 and M6 groups compared with M2 and M3, respectively. These results demonstrated that AST, BA, and yeast complex enhanced the immune response of crabs and were associated with the pathogen defense system.

For crustaceans, the antioxidant capability of hepatopancreas is vital for their health status (50). High levels of ROS will cause oxidative damage to tissues (51), and antioxidant enzymes, such as SOD, GPx, and the lipid peroxidation product MDA, are commonly used as indicators of antioxidant capacity (52, 53). SOD catalyzes the process of superoxide dismutation, and hydrogen peroxide is decomposed into water and oxygen by GPx (54). Moreover, GSH can directly scavenge ROS and becomes oxidized glutathione (GSSG) (55). In this study, we observed that the activity of SOD and GSH contents were significantly increased in M4 for both male and female crabs, indicating that the combination of *R. officinale* Bail, *S. baicalensis*, and *P. orientalis* exhibited better antioxidant effects than that of the M1-M3 groups, which may be attributed to the natural components of these medicinal plants. Previous studies demonstrated that anthraquinone extract from rhubarb can resist oxidant stress in many aquatic animals (56–59). Similarly, the chemical constituents of *S. baicalensis*, including baicalein and polysaccharide, have also shown antioxidant capacity in koi carp (*Cyprinus carpio*) (60), GIFT tilapia (*Oreochromis niloticus*) (61), and *M. rosenbergii* (23). Moreover, activity of SOD and GSHcontents in female crabs and the activity of GPx both in male and female crabs were significantly improved in M5, M6, and M7 treatment groups compared with M2, M3, and M4, respectively. However, the trend of hepatopancreatic MDA level, a marker of endogenous oxidative damage (62), showed an inverted tendency in male crabs. Consistently, these findings suggested that medicinal plant mixtures combined with AST, BA, and yeast complex might promote synergistic effects on the antioxidant capacity of crabs. As feed additives, dietary supplementation with AST, BA, and yeast complex have shown positive effects on the antioxidant function in animals. The antioxidant activity of AST has been reported in coral trout (*Plectropomus leopardus*) (63), loach (*Paramisgurnus dabryanus*) (64), *E.sinensis* (27), red swamp crayfish (*Procambarus clarkii*) (65), and pufferfish (*Takifugu obscurus*) (66). Moreover, BA supplementation also has been reported to increase antioxidant capacity in animals (67, 68). The antioxidant properties of yeast have been reported to improve bacterial resistance in aquatic animal diseases (30, 69, 70). The research of Zhang et al. demonstrated that feeding gibel carp with yeast culture improved their plasma SOD activity after exposure to *A. hydrophila* (71). Similarly, Zhang et al. found that dietary yeast extract supplementation increased CAT and SOD activities in *E. sinensis* (31). Collectively, these results further verified that dietary supplementation with mixtures of *R. officinale* Bail, *S. baicalensis*, *P. orientalis*, AST, BA, and yeast complex is beneficial for improving antioxidant capacity of *E. sinensis*.

Furthermore, combined with the histological characteristic analysis of the hepatopancreas, we observed that the hepatopancreatic tubular structure was improved in the M7 group, which exhibited fewer vacuolar structures and thickened basal lamiae. These results further demonstrated that medicinal plants together with AST, BA, and yeast complex could maintain the health of crab hepatopancreas.

In conclusion, although it is difficult to clarify the mechanism of the synergic action due to the multiple effective ingredients contained in medicinal plants, the present study demonstrated the positive effect of the combination of medicinal plant mixtures and immunostimulants on the immunopotentiation and antioxidation in crabs. In comparison to chemical drugs, herbal preparations have the potential to enhance the growth performance and nutritional quality of aquatic animals, while simultaneously safeguarding their tissue structure. Furthermore, they offer the additional benefits of low cost, low drug resistance, and straightforward operation, which collectively make them an attractive proposition for application in aquaculture. This study provided a feasible formulation of medicinal plant mixture containing 2.5 g/kg *R. officinale* Bail, 1.5 g/kg *S. baicalensis*, and 1.0 g/kg *P. orientalis*, in combination with 600 mg/kg AST, 1.0 g/kg BA, and 20 mg/kg yeast complex, which significantly enhanced the non-specific immunity, antioxidant capacity, and hepatopancreatic health of *E. sinensis*. In future, research should be conducted to investigate more possible combinations of feed additives that are suitable for *E. sinensis* with bio-safety concerns.

## Data Availability

The raw data supporting the conclusions of this article will be made available by the authors, without undue reservation.
